# Diauxic Inhibition: Jacques Monod's Ignored Work

**DOI:** 10.1007/s10739-021-09639-4

**Published:** 2021-05-11

**Authors:** Pierre Louis Blaiseau, Allyson M. Holmes

**Affiliations:** Sorbonne Université, CNRS, UMR7238, Institut de Biologie Paris-Seine, Laboratory of Computational and Quantitative Biology, 75005 Paris, France

**Keywords:** Jacques Monod, Diauxic inhibition, Enzymatic adaptation, Bacterial growth, André Lwoff, Boris Magasanik

## Abstract

Diauxie is at the origin of research that led Jacques Monod (1910–1976), François Jacob (1920–2013), and André Lwoff (1902–1994) to win the Nobel Prize in Physiology or Medicine in 1965 for their description of the first genetic regulatory model. Diauxie is a term coined by Jacques Monod in 1941 in his doctoral dissertation that refers to microbial growth in two phases. In this article, we first examine Monod’s thesis to demonstrate how and why Monod interpreted diauxie as a phenomenon of enzyme inhibition or suppression of adaptive enzymes. We also briefly investigate prior enzyme suppression studies, before Monod’s work, which indicate that he is the first person to observe diauxic growth. Second, we analyse Monod’s post-thesis publications throughout his scientific career, revealing that diauxic inhibition was a significant part of Monod’s scientific activities and greatly fascinated Monod until the end of his life. Paradoxically, Monod’s work and interest on diauxic inhibition are still neglected in historical recounts, focused mostly on Monod’s enzymatic adaptation studies. Indeed, we uncovered a statement by Monod’s colleague, Lwoff, who transformed a quotation from Monod by replacing the word *phenomenon* with *enzymatic adaptation*, which we believe has influenced historians. Finally, we offer hypotheses to explain why Lwoff altered Monod’s statement.

## Introduction

On October 15, 1965, Jacques Monod, François Jacob, and André Lwoff, three biologists comprising a research team at the Pasteur Institute in Paris, received the Nobel Prize in Physiology or Medicine for their discoveries concerning genetic control of enzyme and virus synthesis. This was the first time that a Nobel Prize was awarded to a French group for work in genetics and biochemistry, considered as modern or state-of-the-art biological disciplines. This coveted prize was awarded for the discovery of the “operon model,” which is a regulatory mechanism consisting of a cluster of genes that function under the control of a single promoter. This discovery, specifically in the case of the *lac* operon in *Escherichia coli*, contributed to the elaboration of a new discipline: molecular biology (Morange 1994; Judson 1996). Diauxie is an important source of their research and is widely believed to contribute to the landmark discoveries that greatly influenced the Pasteur trio leading towards their 1965 Nobel Prize achievement (Morange 1994; Judson 1996; Loison 2013; Carroll 2013; Fry 2016; Schwartz 2017).

Diauxie is a term that describes an unusual type of bacterial growth; it is comprised of two phases, separated by an arrested growth period or lag-phase. This double phase growth was discovered by Monod in 1940, as documented in his thesis. To better understand the importance of diauxie in Monod’s research, we recount Monod’s studies during his thesis and analyze Monod’s articles concerning diauxie during and up to the end of his scientific career (1940–1976).

First, we analyse in depth the second section of Monod’s dissertation, which is entirely devoted to diauxie. After the description of diauxie in growth media containing certain combinations of two sugar sources, Monod examined the nature and the mechanism of this strange phenomenon. Based on his experimental results Monod demonstrated that diauxie is caused by the inhibitory action of certain sugars, such as glucose, on adaptive enzymes (meaning an enzyme that appears only in presence of its substrate). Our analysis indicates that while Monod considered diauxie to involve both adaptation and suppression mechanisms, it was the phenomenon of enzyme suppression that most preoccupied him, as described in the introduction of his dissertation:

The second section is devoted to the particular study of a phenomenon to which I have given the name "diauxie," because it expresses growth in the form of two successive outbreaks separated by a phase of zero growth. Without wishing to anticipate the conclusions of this work, we can indicate that this phenomenon is linked to a variation in enzymatic power that occurs in the presence of certain carbohydrate mixtures. More precisely, it seems that it is a phenomenon of enzyme suppression. (Monod 1942, p. 2)

Within the *Discussion* section of the chapter *Le mécanisme de la diauxie*, Monod cited as precursors of enzyme suppression by glucose, the previous works of Frederic Diénert (1900) and Marjory Stephenson’s team (1936–1937). In order to evaluate the place of these studies in Monod’s hypothesis of diauxie, we will compare the results obtained by these authors with those in his thesis. Following Monod, our analysis indicates that these researchers describe examples of enzyme suppression, but they do not observe diauxie. Therefore, we believe that Monod deserves credit for identifying the diauxie double growth phenomenon.

Second, we examine Monod’s writings concerning diauxie after submitting his thesis. During the 1940s, Monod performed new experiments on diauxie and proposed a model of enzyme formation that explained the diauxic inhibition/suppression mechanism. In this model, diauxic inhibition could result from a competition between different sugars for a common precursor of adaptive enzymes. However, during the 1950s, Monod and his coworkers demonstrated that the β-galactosidase enzyme is synthesized de novo without requiring a precursor. Although these results forced Monod to give up his common precursor model, he still believed that the mechanisms underlying diauxie remained “one of the most important aspects of the problem of enzyme biosynthesis” (Monod and Cohn 1952, p. 83). Therefore, he proposed new hypotheses concerning the mechanism of diauxic inhibition by glucose in his successive models of enzyme formation. However, in contrast to his studies in the 1940s, Monod did not publish experimental results in the 1950s pertaining directly to diauxic inhibition. We offer hypotheses for why, during this period, Monod focused his experimental work on the induction of β*-*galactosidase rather than on its suppression by glucose. We further discuss the potential impact of the operon model, developed from 1959 to 1961 with his collaborators François Jacob and Arthur Pardee (1921–2019), on his glucose inhibition studies, for which diauxie is one example. Finally, we show that in the last ten years of Monod’s career, he and his close collaborator Agnès Ullmann (1927–2019) published a few articles about the glucose effect (catabolite repression), using diauxie systems. Taken together, the analysis of Monod’s publications demonstrate that Monod remained interested in the phenomenon of diauxic inhibition throughout his entire scientific career, as shown in his posthumously published article (Ullmann et al. 1976).

In our article the use of primary sources from Monod allowed us to demonstrate that even though he believed that diauxie involved adaptive or induced enzymes, he also strongly believed throughout his scientific career that the problem of diauxie was due to the inhibition of these enzymes by sugars such as glucose. This indicates that from Monod’s point of view, the study of diauxie could not be solely restricted to the question of enzyme adaptation. On the contrary, many historical sources recount that Monod’s work, from his thesis to the Nobel prize, was exclusively based on enzymatic adaptation/induction. There is, as far as we can tell, little mention of his interest and work on diauxic inhibition. To better reconcile and balance these viewpoints, we include the role played by Monod’s mentor Lwoff, who honored Monod in his obituary tribute but also changed one of Monod’s spoken phrases delivered in his Nobel Prize acceptance speech (Monod 1966; Lwoff 1977). Lwoff deliberately replaced Monod’s original use of the word “phenomenon” with “enzymatic adaptation.” We discuss why Lwoff intentionally made this word substitution that subsequently influences historical accounts of Monod’s scientific work.

## Part One: Monod’s Dissertation and the Discovery of Diauxic Growth

Since 1937, Monod had shown an interest in studying the growth of bacterial populations using rich media (Monod 1937). During work on his thesis, he switched from rich media to the use of synthetic growth media, which enabled him to add unique carbon sources, such as glucose or sucrose, in order to monitor their effects (Monod 1941). This was important because it allowed him to accurately calculate what he termed *growth coefficients*, such as yield and growth rates. On the first page of his doctoral dissertation, *Research on the Growth of Bacterial Cultures,* defended on December 17, 1941, Monod defined the objective of this research:

We hope to show in this presentation that the quantitative study of growth makes it possible to experimentally demonstrate certain problems that have hardly been addressed in bacterial physiology thus far: in particular the influence of the chemical structure of substrates on the activity and yield of synthetic reactions. (Monod 1942, p. 2)

Monod rightly assumed that the mathematical quantification of growth would further allow him to go beyond his expectations and identify a new biological phenomenon: diauxie.

In the first part of his thesis, Monod showed that the bacterial growth rate values vary according to the nature of the sugar added to the synthetic growth medium. The question then arose as to the relationship among the enzymes that degrade these sugars, specifically, whether they are dependent on each other or act separately. In an attempt to answer this question, Monod quantified the growth rate in a medium containing two sugar sources. It was during this experiment that he observed an unexpected result:

In studying mixtures of sucrose and dextrin, I was surprised to find that growth in this case was expressed in a completely aberrant manner: it was separated into two quite distinct growth rates, together comprising all the phases of a normal growth curve. (Monod 1942, pp. 140–141)

Monod was thus fully aware that he had discovered a new biological phenomenon that no one had previously observed. He coins the term *diauxie* to explain this phenomenon and considers it important enough to devote the second and last part of his thesis to describe diauxie. As he noted:

The second part of this work is devoted to the study of a complex growth phenomenon that occurs in bacterial cultures in the presence of certain carbohydrate mixtures. I apologize for having to designate this phenomenon by creating a new term: that of "diauxie" (double growth). The rest of this presentation will, I hope, convince you that this is indeed a new and defined problem, which needed to be distinguished by a special term. (Monod 1942, p. 139)

The first two chapters explain and explore diauxie.

In the first chapter, entitled *Le phénomène de diauxie*, Monod described results obtained by a systematic study using many combinations of sugars and three different bacteria, comprised of *Bacterium (Escherichia*) *coli*, *Bacillus subtilis*, and *Bacillus typhimurium (Salmonella enterica).* His work showed that the phenomenon of diauxie is restricted to certain mixtures and it classified sugars according to their behavior in the presence of glucose, either *A* sugars which do not cause diauxie, or *B* sugars which give rise to this phenomenon.

**Fig. 1:**
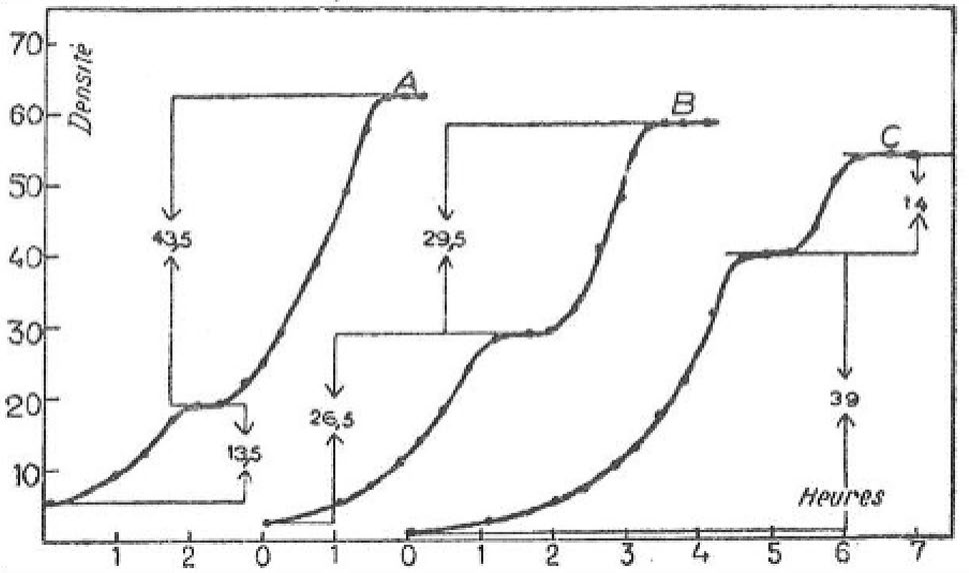
Growth of *Bacterium coli* in a mixture of glucose-sorbite, in the following proportions 1/3 (*A*); 2/2 (*B*), and 3/1 (*C*). Adapted from Fig. 51 in Monod’s thesis (Monod 1942, p. 167)

In the second chapter, *La nature du phénomène de diauxie,* Monod described how variation in the concentrations of *A* and *B* sugars impacted the outcome. He demonstrated that total growth at the end of the first cycle always changed in proportion to the concentration of the *A* sugar added to the culture. Likewise, the second growth cycle always varied in proportion with the concentration of the *B* sugar. Monod thus concluded that the *A* sugar is fully utilized during the first cycle of growth and that subsequently the *B* sugar is used during the second cycle of growth. If we consider, following Monod, the examples of different proportions of glucose (*A* sugar) + sorbite (*B* sugar), we see how Monod demonstrated that glucose is entirely consumed at the end of the first growth cycle (Fig. 1). To confirm this hypothesis, Monod used a direct dosage method to quantify the amount of reducing sugars in a mixture of glucose + sorbite in which glucose is the sole reducing sugar. Taken together, these two experiments allowed Monod to demonstrate clearly that sugars in the *A.B*. mixture are used successively or step-wise: first *A* then *B*.

To better orient his research, Monod then discussed his various experimentally-based hypotheses to explain diauxie. He refutes the vague notion of "preferential attack," an argument used since the time of Louis Pasteur to describe a mixture of optical isomers, which Monod believed was not relevant to help understand diauxie. Above all, the question for Monod was not related to a *preference* for sugar but rather to the inability of sugars *A* and *B* to be used simultaneously. For Monod, the diauxie question corresponded to the variation of what he called *enzymatic power*. In this case, then, two classical mechanisms could explain diauxie: either selective genetic mutation or enzymatic adaptation. Interestingly, at this point Monod also forwarned the reader that there may also be a third explanation that could account for diauxie:

Thus, everything happens as if, during the first outbreak of a diauxie, the culture acquired and utilized the enzyme corresponding to sugar A, but did not acquire or did not form in sufficient quantity the enzyme corresponding to sugar B. Diauxie appears to be the expression of a variation of enzymatic power. When it comes to explaining such a variation, two explanations are always present: adaptation or selection…. It remains to be seen whether one of these two hypotheses is the right one, or whether it will be necessary to resort to another type of explanation. (Monod 1942, pp. 170, 172)

To test the adaptation and genetic mutation selection hypotheses further, Monod then performed a series of experiments to "adapt" bacteria to the *B* sugar before placing it in a mixture of the two sugars. Monod’s experimental results showed that (1) diauxie is not eliminated by preadaptation to growth in the *B* sugar, and (2) diauxie occurs even if the *A* sugar is added when the consumption of *B* sugar has already begun. Therefore, Monod concludes that neither genetic mutation selection nor adaptation can fully account for diauxie. Rather, he insists that adaptation is “powerless” to explain all aspects of this phenomenon:

It is therefore impossible to completely suppress diauxie by prior adaptation by growth in the B sugar…. Therefore, we must admit that the “adaptive” hypothesis is powerless to explain all of the effects we observe concerning diauxie…. Experience, however, shows that the hypothesis of selection is unsustainable, and that the hypothesis of adaptation is in any case insufficient; indeed, one can neither suppress nor reverse the diauxie in a mixture A. B. by prior cultivation of the strain in the presence of B sugar as the only carbon food. It is also impossible to induce diauxie in a mixture of two B sugars by prior training with one of these two sugars. However, it seems that adaptation plays a certain role in the phenomenon. If it cannot suppress the diauxie in a mixture A. B. it can at least attenuate it*.* (Monod 1942, pp. 178, 183)

In the third and last chapter devoted to diauxie, *Le mécanisme de la diauxie*, Monod noted that his classification of *A* and *B* sugars correlates with the enzymatic classification established by the Finnish biologist Henning Karström (1899–1989) distinguishing constitutive and adaptive enzymes:

This author (Kartström, 1930) showed that one could, in the bacterial enzymatic arsenal, distinguish between two enzymatic categories: those (constitutive enzymes) that are aways present, regardless of the culture media composition; the others that appear in an appreciable quantity only when the culture media contains the appropriate substrate (adaptive enzymes)…. Anyway, an evident correlation exists between this classification and that which allows to establish the phenomenon of diauxie: the A sugars seem to correspond with the constitutive enzymes, the B sugars to the adaptive enzymes. (Monod 1942, pp. 171, 186)

The correlation Monod established confirms a link between diauxie and the phenomenon of adaptation. However, as shown above, Monod considers that the adaptive hypothesis is not sufficient by itself to account for the mechanism of diauxie. For this reason he developed an additional hypothesis to explain this phenomenon: the inhibitory action of *A* sugars.

To evaluate the inhibitory action of *A* sugars, Monod performed a new type of experiment. By using a bacterial culture preadapted to growth in a *B* sugar, Monod showed that subsequent growth of the same culture in presence of the *A* sugar, even for a short time, suppresses the adaptation to growth in the *B* sugar. Thus, Monod conclusively demonstrated that an *A* sugar can suppress growth adaptation to a *B* sugar when this adaptation is already acquired. As Monod explained this:

It is therefore becoming increasingly clear that diauxie is linked to a phenomenon of adaptation. But then an essential fact remains to be explained: that diauxie cannot be suppressed by any prior adaptation and that it occurs even when an A sugar is added in a culture already in the process of active proliferation at the expense of a B sugar. It is now more or less obvious that this fact can only be explained by the inhibitory action of A sugars…. Repeated, using the same conditions with other sugars, this experience gave identical results. The interpretation leaves no doubt: in the presence of the A sugar (some of them only in *Bacterium coli* cases), the adaptation to sugar B is inhibited. Adaptation is suppressed if it is already acquired. (Monod 1942, pp. 189, 190–191)

Because of this experimental result, Monod preferred to use the term *suppression,* which he believes is a more precise term than *inhibition*, to define the action of the *A* sugar. However, Monod did not make a clear distinction between suppression and inhibition in the case of diauxic growth conditions where both A and B sugars are added simultaneously. Therefore, Monod used both *inhibition* and *suppression* in his thesis when referring to diauxie.

Within the *Discussion* section of chapter three and, finally, in the *Summary and Conclusions* part of his thesis dedicated to diauxie, Monod repeatedly insisted that diauxie must be considered as a phenomenon of inhibition or suppression of adaptive enzymes:

All the results that have just been presented indicate that diauxie is the expression of a variation in enzymatic power and that this variation is related to the adaptive nature of certain enzymes and to the power that certain sugars seem to have to inhibit these enzymes, or more precisely to inhibit their adaptation. What is the nature of this inhibition, its precise mechanism—such is the problem ultimately posed by the phenomenon of diauxie. (Monod 1942, p. 193)

To explain the phenomenon of diauxie, one must account for both the mechanism of adaptation and that of suppression. Any theory about the formation of bacterial enzymes will have to take into account these facts, in particular the inhibitory property of certain substrates*…*.

Under these conditions it seems certain that the phenomenon expressed by the diauxie curves is an inhibition or rather a suppression of enzymes: in the presence of certain sugars (inhibitory sugars) the adaptation to B sugars is inhibited. The existence of such a phenomenon has already been reported by some authors whose conclusions are in agreement with those of the study of diauxie….

A few cases of enzyme suppression have been reported, but the mechanism of this phenomenon is unknown. The general facts revealed by the study of diauxie demonstrate its importance in bacterial physiology and help to clarify the problem. However, it is not yet possible to propose a precise explanatory scheme. Any theory on the mechanism of enzyme formation in microorganisms will have to take these facts into account and be able to explain them*.* (Monod 1942, pp. 193, 195, 197, 199)

Thus, Monod understood that he had discovered a novel growth phenomenon that could also have a major impact on understanding how enzymes are formed, a major question that concerned biologists at this time. For this reason, we can anticipate that Monod will continue to work fervently on the mechanism of diauxie.

## Monod’s Predecessors

Towards the end of his thesis, Monod cites “cases of enzyme suppression” that had been published prior to his own work. These included the studies of the French Pastorian Frederic Diénert and the English team led by Marjory Stephenson:

The first observation of this phenomenon seems to be attributed to Diénert (1900). He showed that yeast, pre-grown first in galactose, partially loses its adaptation in the presence of glucose…. Stephenson and Yudkin (1936) studied this case with precision. (Monod 1942, pp. 193, 194)

References to their publications were often mentioned by Monod in his later papers (Monod 1947, 1966). Furthermore, Monod’s reference to Diénert is sometimes recounted, not so much for Diénert's observation of an enzyme suppression effected by glucose, but as a precursor to Monod's own discovery of the diauxic effect. As Loison noted:

Just as Monod would do 40 years later, Diénert observed that fermentations of two different sugars (glucose and galactose, for example) were not independent. If one existed, it slowed the speed of the second…. Monod rediscovered the phenomenon that, in 1900, Dienert had already observed in yeasts: the presence of some specific sugars (glucose, fructose, saccharose, mannite, and mannose) inhibits the fermentation of other sugars by the bacterium*.* (Loison 2013, pp. 170, 174)

Therefore, to understand the relevance of Diénert and Stephenson’s team for Monod’s study of diauxie, it is important to investigate and compare their cited works to the description found in Monod’s thesis.

### Diénert’s Thesis (1900)

Frédéric Diénert (1874–1948), a representative of the third generation of Pasteurians, was a student of Emile Duclaux (1840–1904), who succeeded Louis Pasteur (1822–1895) as the head of the Institute he had founded in 1888. The work that is cited by Monod concerning the glucose effect was discussed in Diénert’s thesis, *Sur la fermentation du galactose et sur l'accoutumance des levures à ce sucre* (1900).

In Chapter 3, *Accoutumance des levures au galactose*, Diénert first showed that yeast must be “habituated” or adapted to galactose before it is able to ferment this sugar. In order to assess the duration and conditions of galactose habituation, Diénert grew cultures in the presence of galactose to accustom the yeast to this sugar, then washed the cells and placed them in a medium containing either galactose or glucose. His results showed that some sugars, such as glucose, cause the cells to partially lose their habituation to growth in galactose. Importantly, according to Diénert’s experimental protocol, glucose and galactose were not mixed together, as Monod did, but were added successively, starting from the same preculture.

In Chapter 6, *Mélange de deux sucres et acclimatation*, Diénert described what happened when glucose and galactose were added together, similar to the diauxic growth conditions performed by Monod (Experiment 1, pp. 173–176). But in contrast to Monod’s interpretation, Diénert concluded that glucose helped yeast to grow by promoting the habituation to galactose:

As we have already seen, the addition of a small amount of glucose to the culture medium restores the vitality of the yeast while at the same time promoting its “habituation” to galactose. (Diénert 1900, p. 177)

Interestingly, in a medium containing a mixture of two sugars, Diénert’s observation does not support and indeed contradicts Monod’s observation of diauxie. This discrepancy could be explained by the fact that Diénert’s growth measurements were imprecise and therefore could not allow him to observe the two growth phases. The difficulties in interpreting Diénert’s data are reported by Stephenson and Yudkin:

These experiments of Diénert, though highly suggestive, are difficult to interpret owing principally to their non-quantitative nature, rates of fermentation, quantity of yeast and change in cell numbers not being recorded. (Stephenson and Yudkin 1936 p. 506)

### Stephenson’s Team Work, 1936–1937

In the early 1930s, two British research groups were investigating the glucose inhibitory effect on bacterial enzymes. The main enzyme models included enzymes involved in amino acid degradation pathways, such as tryptophanase, studied by Charles Happold's (1902–1991) team at the University of Leeds (Happold and Hoyle 1936), and deaminases, studied by Marjory Stephenson's (1885–1948) team at Cambridge (Stephenson and Gale 1937a). The results of this research were interpreted using the hypothetical framework of the "protein sparing action" of sugars, first proposed in 1912 by the American bacteriologist Arthur Kendall (1877–1959). According to this hypothesis, derived from the old idea of the antagonism between fermentation and putrefaction, the metabolism of sugars would spare protein usage for the production of energy:

A most fundamental principle of bacterial metabolism may be expressed concisely by stating that “Fermentation takes precedence over putrefaction;" that is to say, bacteria in general which can utilize both carbohydrate and protein, act upon the former in preference to the latter when both are present in the same medium*.* (Kendall and Farmer 1912, p. 63)

Parallel to their studies of the inhibitory effect of glucose on deaminases, Stephenson and her collaborators, John Yudkin (1910–1995) and Ernest Gale (1914–2005), developed a line of research to understand the problem of enzymatic adaptation. During work on enzymatic adaptation to galactose, Stephenson and Yudkin showed that glucose had a suppressive effect on galactozymase activity in yeast cells when glucose is added to a preadapted yeast culture in media containing galactose. As they explained, “In confirmation and extension of earlier work we find that adapted cells lose their galactozymase completely after fermenting glucose and regain it in the presence of galactose” (Stephenson and Yudkin 1936, p. 514).

The following year, Stephenson and Gale presented a similar study on enzymatic adaptation to galactose in the bacteria *E. coli*. In this study, they quantified the inhibitory effect of glucose on the production of galactozymase in a medium containing a mixture of galactose and glucose added together at the beginning of the growth experiment (Stephenson and Gale 1937b). Thus, similar to Monod, they measured enzyme inhibition by glucose in *E. coli* cultures and in a mixture of *A*:*B* sugars. However, they did not measure cellular growth but only the indirect presence of galactozymase. Moreover, unlike Monod, they did not use a synthetic growth medium that allowed him to interpret his experimental results and to discover the double growth phases of diauxie.

In summary, Diénert observed an inhibitory effect of glucose on galactose consumption, but only when these sugars were added successively and not simultaneously. Stephenson’s team revealed an inhibition of galactozymase activity by glucose in a medium containing a mixture of glucose and galactose, but they did not observe the double growth pattern that characterizes diauxie. These comparative analyses indicate that Monod was the first scientist to put all of the pieces of the puzzle together by interpreting his experimental results as the diauxie phenomenon.

## Part Two: Monod and Diauxic Inhibition, 1943–1976

In order to explain the formation of adaptive enzymes, Yudkin had described a model in 1938 explaining kinetic enzyme formation called “mass action theory.” According to this model, each adaptive enzyme should be in equilibrium with one or more of its precursors. Combining the substrate with the part of the enzyme that is already present should then induce the production of more enzymes to restore the precursor-enzyme balance (Yudkin 1938).

**Fig. 2:**
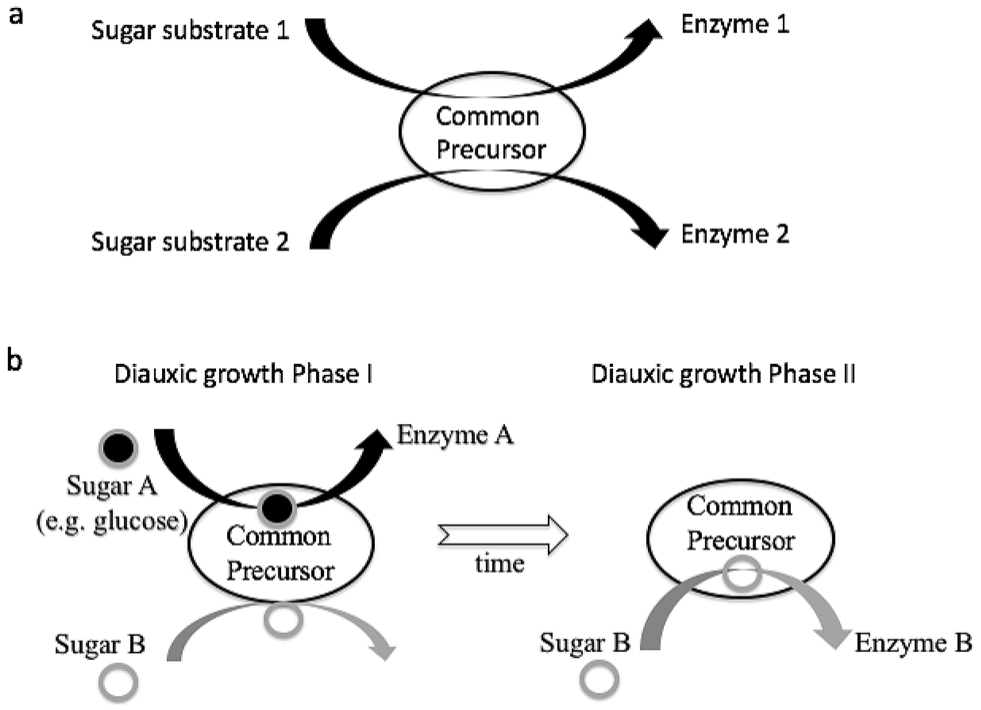
Schematic interpretations of **a** Monod’s common precursor model and **b** diauxie. **a** This model postulates the existence of a common precursor capable of interacting with different sugar substrates to form specific enzymes. **b** Competition between sugars *A* (e.g., glucose) and *B* for interaction with a common precursor is depicted in the first diauxic growth phase in which only sugar *A* binds efficiently. When sugar *A* is depleted during a time of growth lag, sugar *B* can subsequently bind to the common precursor in the second diauxic growth phase

In 1943, Monod proposed an alternative model of enzyme formation to explain both enzymatic adaptation and diauxic inhibition. While Monod retained Yudkin's idea of an enzyme precursor, in his model the precursor became common to several enzymes, which only formed after the interaction of the common precursor with one of the sugar substrates (Fig. 2a). This model had the advantage of explaining diauxie by reconciling the two contradictory aspects of this phenomenon: the specificity of adaptation to a given sugar and the more general effect of the inhibition of adaptive enzymes (Fig. 2b). As he explained it:

But, to be complete, a schematic of the adaptation mechanism must at the same time explain the characteristic inhibition of diauxic growth. One could therefore imagine that the series of enzymes attacking carbohydrates possess a common precursor, able to recognize these bodies with a weak but general affinity, and capable, in the presence of some of them, of acquiring a specificity, i.e., a closer adaptation. The monopolization of the precursor by certain substrates could then explain the diauxic inhibition. (Monod 1943, p. 181)

In 1945, Monod carried out experiments to test his competitive sugar usage model of the common precursor, in which he varied the ratio of the concentrations of sugars *A* "constitutive" and *B* "adaptive." The results showed that the level of diauxic inhibition can be reduced and even eliminated by increasing the concentration of the *B* sugar relative to the *A* sugar. These results appeared to be consistent with the model of *A* and *B* sugars competing for a common precursor. As Monod expressed this:

In any case, the results that have just been presented show that diauxic inhibition has the essential character of a competitive phenomenon and that seems to depend on the relative affinity of the two constituents of an A-B mixture for the same surface or, if one prefers, for the same molecule. (Monod 1945, pp. 39–40)

In a 1947 review of enzymatic adaptation, Monod devoted 11 out of 65 pages to the phenomenon of diauxie. In this he described the results of his experiments in great detail, commenting on nine figures and presenting his explanatory model of the common precursor. As he noted:

Whatever may be the mechanism of these interactions, it is obvious that their occurrence points to a common, or at least a partly common origin of different, specific molecules. The simplest way to express this is to admit that a common precursor or a common pool of precursor molecules (building blocks) is involved in the synthesis of the interacting enzymes. (Monod 1947, p. 259)

Thus, by the end of the 1940s, both enzymatic adaptation and diauxic inhibition were studied by Monod in the context of his common precursor model in order to understand the fundamental question of enzyme formation. This was described by his closest collaborator during this period, the Italian researcher Annamaria Torriani (1918–2013):

The core of the question in those days (1948–1950) was to understand why there was an increased rate of enzyme formation upon addition of the substrate (adaptation) or a diauxic inhibition. The working hypothesis in the lab was that “many different enzymes may stem from a common precursor or pool of precursor molecules.” (Torriani 1980, p. 44)

However, enzymatic adaptation studies required more refined experimental approaches. Thus, new experimental tools were devised to test the validity of the common recursor model.

The enzyme β-galactosidase (called lactase) that was extracted and purified from *E. coli* in 1948 became Monod's preferred enzyme model (Monod et al. 1948). Indeed, a fluorescent substrate, orthonitrophenyl-β-D-galactoside (O.N.P.G.), discovered by the American researcher Joshua Lederberg (1925–2008), made it easy to measure the specific activity of β-galactosidase in a very sensitive way (Lederberg 1950). Enzymatic adaption studies also required more precise theoretical concepts. In 1953, Monod, Melvin Cohn (1922–2018), his colleague on the study on enzyme adaptation since 1948, along with others, published “Terminology of Enzyme Formation,” in which the term *enzymatic adaptation* was replaced by *enzymatic induction*. The authors explained that the term *adaptation* had a broader sense, in biological parlance, than just corresponding to the formation of enzymes specifically induced by their substrates or chemical analogues:

The term (enzymatic adaptation) was, perhaps, an unfortunate choice since the word “adaptation” has an old and well established meaning…We therefore propose the following terms and designations; previously used terms are placed in parenthesis. A relative increase in the rate of synthesis of a specific apo-enzyme resulting from exposure to a chemical substance is an “enzyme induction” (enzyme adaptation). Any substance thus inducing enzyme synthesis is an enzyme “inducer.” (Cohn et al. 1953, p. 1096)

Now poised with the ability to measure β-galactosidase with sensitivity, Monod then tested his favorite common precursor model. He measured this enzyme activity by using auxotrophic mutant strains of *E. coli* that were unable to grow in the absence of certain amino acids. His results revealed that enzyme synthesis and bacterial cell division were strictly correlated and depended on the addition of amino acids to the culture medium. Monod was surprised by these results because they suggested that β-galactosidase was synthesized de novo from assembled amino acids, without the need for a precursor. As he noted: “By its very simplicity, the linear relationship found between the induced synthesis of the enzyme and the synthesis of a new living substance is surprising” (Monod et al. 1952, p. 659).

In 1955, radioactive pulse-chase labeling experiments further confirmed Monod’s results and definitively showed that the biosynthesis of an enzyme is made de novo from newly assembled amino acids. The induced production of the β-galactosidase enzyme is, therefore, not made from the conversion of a precursor:

The incorporation data demonstrate conclusively that the induced synthesis of β-galactosidaseinvolves the complete de novoformation of the molecule from the elements. The hypothesis that the induced enzyme is a conversion product of another protein, formed and accumulated in the absence of inducer, is eliminated. (Hogness et al. 1955, p. 112)

Thus, Monod's own experimental results contradicted his common precursor model of enzyme formation. Consequently, diauxic inhibition could not be explained by this model. Nevertheless, Monod was still intrigued by this phenomenon. As described in his paper with Cohn in 1952, studying diauxie required using new techniques:

The mechanism of diauxie, is no better understood today than it was 10 years ago. The observations made in bacteria and yeast do not allow clear understanding of the level of interactions. In conclusion, the question of interactions, that of the mechanism of diauxie and that of the in vivo stability of enzymes and their "reconversions" should be taken up again using techniques that would make it possible to ask these questions in a very precise form*.* (Monod and Cohn 1952, pp. 81, 83)

Already in 1953, Monod and Cohn had proposed a unitary model of enzyme formation in which induction is presented as a universal phenomenon for all "adaptive" and "constitutive" enzymes. In this generalized induction model, the induction of the constitutive enzymes exists but is masked. Within the context of this new generalized induction model, Monod tried to explain diauxic inhibition by employing a mechanism in which glucose would block the transport of inducing sugars into the cell:

A type of inhibition of exogenous induction (which we call the “glucose diauxic effect”) is exerted by a variety of carbohydrates, among which glucose is by far the most potent…. Its mechanism is not clear, but it is conveniently described by the assumption that glucose, even at remarkably low concentrations, is able to prevent the penetration of certain carbohydrate-inducers into the cell. In other words, the glucose “diauxic effect,” whatever it is, appears to be exercised on the earlier, if not on the very first step, of the “inducer metabolism.” (Cohn and Monod 1953, p. 137)

However, in 1956, this explanation for diauxic inhibition was called into question by work performed at Harvard University by the American biochemist Boris Magasanik (1919–2013). Studying the bacterium *Aerobacter aerogenes* (*Klebsiella aerogenes*), Magasanik, along with his PhD student Frederick Neidhardt (1931–2016), showed that the presence of glucose does not prevent the uptake of galactose. Evidence was provided by measuring the quantity of both sugars in culture cells using the recently updated method of paper chromatography (Neidhardt and Magasanik 1956).

In response, in 1958 Monod postulated a revised version of his generalized induction model, whereby the existence of an inactive pre-enzyme could be converted into an active enzyme by combining with an inducer. This model assumed that the interaction of a pre-enzyme with the inducer required the inducer to undergo an activation step. Monod justified this extra step in order to explain the phenomenon of diauxic inhibition, which supposed that glucose competed with some unknown factor needed to activate the inducer:

The interest of this admittedly very speculative assumption is to offer perhaps an interpretation of one of the most conspicuous and general effects encountered in inducible systems. I mean the inhibitory action of glucose and other carbohydrates in induction. As it is well known, this effect has been observed in a wide variety of systems, and is the cause of diauxic growth…. It is tempting to suppose that glucose or other carbohydrates may compete with many different substances for a common activating step which might be required for an effective interaction of the inducer and the enzyme-forming center. (Monod 1958, pp. 582–583)

In summary, analysis of Monod's publications in the 1940s and 1950s shows that diauxic inhibition remained an essential part of his models describing enzyme formation. Monod’s hypotheses concerning the role of the inhibitory effect of glucose evolved in tandem with his enzyme formation models. First, in the common precursor model, glucose is in competition with other sugars for the ability to interact with the precursor; second, within different versions of his generalized induction model, glucose can either block the transport of the inducer or prevent the activation of a pre-enzyme (Monod 1943, 1958; Cohn and Monod 1953).

In the 1950s, Monod proposed different models but did not publish experimental results directly pertaining to diauxic inhibition, as he had in the 1940s. It appears that the rejection of his common precursor model may have had important consequences for his diauxie studies. This model, though ultimately unsuccessful, was able to explain both enzymatic adaptation as well as suppression by glucose. Both of these components of diauxie revolved around the same mechanism involving conversion of a common precursor into a series of enzymes that are subsequently induced (Fig. 2b). This made diauxie very attractive for studying the problem of enzyme formation. After the rejection of the initial common precursor model, Monod offered alternative models in 1953 and 1958. In Monod’s model of 1953, the role of glucose, proposed to inhibit the transport of an inducer, was contradicted by the work of Magasanik. Monod’s 1958 model, however, was admittedly speculative, and it depended on unknown variables, making the explanation of diauxic inhibition difficult to test. Taken altogether, and positioned within a very competitive research field, Monod probably preferred to concentrate his experiments on exploring the specific induction of β-galactosidase.

In order to understand the general suppressive effect of glucose on several enzyme systems, Monod’s main competitor, Magasanik, concurrently developed a comprehensive metabolic approach to analyze enzymes inhibited by glucose that participate in the catabolism of many amino acids and sugars. In 1961, Magasanik proposed the general model of "catabolite repression." It assumed that glucose degradation produces catabolites very rapidly. These catabolites, in a feedback loop, repress the formation of other enzymatic systems that produce the same catabolites as produced by glucose degradation, but more slowly. This physiological model accounts for all known examples of the enzymatic inhibiting effect of the glucose, including diauxie (Magasanik 1961, p. 251).

From 1961 on, researchers, including Monod, considered the phenomenon of diauxic inhibition as one example of catabolite repression (also called “glucose effect”). The same year, Monod published with François Jacob the genetic regulatory operon model (Jacob and Monod 1961). This paper marked the end of what is considered the “glorious period” (1959–1961) of collaboration between Monod, Jacob, and Arthur Pardee that led to new genetic concepts and terms such as repressors, operon, and messenger RNA. In parallel with his operon work, and undoubtedly influenced by it, Monod performed preliminary genetic studies to investigate whether the glucose effect also depended on the same genetic factors necessary for the regulation of the *lac* operon (Brown and Monod 1961). Monod and Jacob interpreted and tentatively concluded that the glucose effect was not perturbed when using *lac* gene mutant strains, and that other genetic factors needed to be identified and characterized in order to better understand diauxic inhibition (Monod and Jacob 1961).

During the 1960s and 1970s, Monod continued to be interested in the phenomenon of diauxic inhibition, this in spite of his considerable administrative duties, especially after becoming head of the Pasteur Institute in 1971. In 1968, Monod and his important collaborator Agnès Ullmann showed that cyclic Adenosine Mono Phosphate (cAMP), and no other adenine nucleotide derivatives tested, was specifically closely linked to “catabolite repression.” The role of cAMP was then evaluated using different diauxic systems. Their results indicated that cAMP suppresses all of the diauxies analyzed (glucose-lactose, glucose-maltose, and glucose-xylose) (Ullmann and Monod 1968). These results, in combination with the discovery in 1970 of the cAMP receptor, called the Catabolite Activator Protein (CAP), highlighted the role of the cCAP-AMP complex as a major element of the “catabolite repression” pathway (Zubay et al. 1970).

In Monod’s posthumous article, Monod with Agnès Ullmann and Françoise Tillier questioned the role of cAMP as the sole regulator of catabolic repression (Ullmann et al. 1976). The search for mediators other than cAMP, using a biochemical approach, allowed them to partially purify a metabolite of low molecular weight, called Catabolite Modulator Factor, which exerts a repressive effect on the expression of several operons subjected to catabolic repression. These results suggested that an independent pathway involving the cCAP-AMP complex controls the activity of enzymes sensitive to “catabolite repression.” These two articles underline the attachment that Monod retained for diauxic inhibition until the end of his scientific career (Ullmann and Monod 1968; Ullmann et al. 1976).

## Conclusion

In this article, we examined the thesis work of Jacques Monod and his views concerning diauxic inhibition throughout his career to claim that he never stopped trying to understand this phenomenon. We attempt to clarify this aspect of Monod’s work, which appears to have been overlooked by historians. Upon careful investigation and analysis of Monod’s thesis as well as his later scientific research concerning diauxie**,** the following conclusions can be drawn: (1) Monod, in his thesis, was the first to observe diauxic growth, which he interpreted as a consequence of enzymatic inhibition of adaptive enzymes; and (2) Monod devoted a significant part of his scientific efforts towards understanding the mechanistic aspects of diauxic inhibition while studying enzymatic adaptation. During the 1940s and 1950s, the subject of diauxie remained important to Monod, as can be seen in his published biochemical models of enzyme adaptation, renamed induction (Monod 1943, 1958; Cohn and Monod 1953). In his later years, Monod’s collaboration with Agnès Ullmann further shows his tenacious efforts towards understanding the glucose diauxic inhibition effect.

Monod’s discovery of diauxie during his thesis is generally acknowledged. Some have even commemorated diauxie as Monod’s seminal work, which led to his further studies on enzymatic adaptation. Monod’s studies then converged with genetic phage studies that, all together, contributed to the first model of genetic regulation, resulting in the award of the Nobel Prize to Monod and his colleagues Jacob and Lwoff in 1965 (Morange 1994; Judson 1996; Loison 2013; Carroll 2013; Fry 2016; Schwartz 2017).

Nonetheless, Monod’s unrelentling efforts towards understanding diauxic inhibition have not previously been fully recognized. This may be for the following reasons. First, Monod’s famous work on the operon model was scientifically groundbreaking and successful, so his diauxic inhibition studies naturally remain in the shadow of this more famous work as relatively secondary and less important. Second, diauxie was linked so tightly to enzymatic adaptation that the term *diauxie* got lost in translation and was replaced by enzymatic adaptation, despite Monod’s attempt to separate the two phenomena.

Finally, there may be an even more precise reason for why Monod’s diauxie story has been buried in the annals of history. We have examined various historical accounts that describe discussions between Monod and his mentor Lwoff in 1940 at the Pasteur Institute. According to most of these accounts, Lwoff suggested that diauxie was a phenomenon of enzymatic adaptation. Monod accepted Lwoff's hypothesis and subsequently devoted his future research activities to work on enzymatic adaptation, as recounted in the following anecdotes:

As he himself recounts in his Nobel lecture: in December 1940, he went to André Lwoff, then head of the microbial physiology department at the Pasteur Institute, to ask his opinion on a new phenomenon, and Lwoff replied that it could be considered as a phenomenon of "enzymatic adaptation" of bacteria. From that day on, wrote Monod, "All my activity was devoted to the study of enzymatic adaptation." (Fantini 1988, p. 11)

Based on the indications given by A. Lwoff, this two-step growth was interpreted as a case of "enzymatic adaptation.” (Gaudillière 1991, p. 15)

Lwoff suggested to Monod that the biphasic growth curves were due to the bacteria, after a lull, adapting to the second, less preferred, food-much like Parisians had to do during that hard, cold winter. How the bacteria adapted was a complete mystery, however, as essentially nothing was revealed by the earlier researchers or known in 1941 about how the activities or production of enzymes were controlled. Monod decided on the spot that this would be his quest: he would get to the bottom of enzyme adaptation. (Carroll 2013, p. 134)

In the years immediately following the Second World War (1944–1947), as Lwoff had suggested, Monod tended to interpret "diauxie" as an example of enzymatic adaptation, as defined by Diénert and Karström. (Schwartz 2017, p. 24)

However, Agnès Ullmann, his close collaborator, emphasized that Monod interpreted diauxie as a phenomenon of enzyme inhibition:

His interpretation of the diauxic growth phenomenon was that glucose (the first sugar used by the bacterium) inhibited the formation of an enzyme necessary for assimilating the second sugar; the latency period between the two growth phases corresponded to the “induction time” of that enzyme*.* (Ullmann 2010, p. 69)

Attempting to understand this contradiction, we compared Lwoff’s statement with Monod’s original Nobel Prize speech. In it, he stated:

Lwoff’s intuition was correct. The phenomenon of "diauxie" that I had discovered was indeed closely related to enzyme adaptation, as my experiments, included in the second part of my doctoral dissertation, soon convinced me. It was actually a case of the "glucose effect" discovered by Diénert as early as 1900, today better known as "catabolic repression” from the studies of Magasanik. The die was cast. Since that day in December 1940, all my scientific activity has been devoted to the study of this phenomenon. (Monod 1966, p. 475)

Monod thus makes clear in his own words that diauxie is attributable to the “glucose effect” and not to enzymatic adaptation, another closely related observation. This nuance is very important because, as Monod states here, he remained faithful to what he understood about diauxie during his thesis twenty-five years earlier. From these carefully chosen words by Monod in his Nobel Prize speech, we believe that the meaning of the word *phenomenon* at the end of this quotation is the same as its use at the beginning, that is, the "phenomenon of diauxie." In this light, Monod stated that he had devoted all of his scientific activity to the study of diauxie and the two aspects of this biological phenomenon: the inhibition and induction of enzymes.

To our knowledge, the first to replace the word *phenomenon* by that of *enzymatic adaptation* was Lwoff in his 1977 obituary of Monod commissioned by the Royal Society:

Jacques Monod told how, in December 1940, at the Institut Pasteur, he came and showed me the diauxic curve and asked: “What could that mean?” I said it could have something to do with enzymatic adaptation. The answer was: “Enzymatic adaptation, what is that?” I told Monod what was known—what I knew—and he objected that the diauxic curve showed an inhibition rather than an “adaptation.” We know today that repression and induction are complementary, but I simply repeated that diauxie should be related to adaptation…. It turned out that glucose was inhibiting the synthesis of a few enzymes responsible for the metabolism of the other sugars-catabolite repression—but the enzymes involved in diauxie were nevertheless “adaptive.” Induced enzyme synthesis was the key to diauxie… “From this very day of December 1940,” wrote Jacques Monod, “all my scientific activity has been devoted to the study of enzymatic adaptation.” (Lwoff 1977, p. 388)

Thus, Lwoff publicly stated that from December 1940, Monod was convinced that enzymatic adaptation (renamed later “induced enzyme synthesis”) was the key to diauxie. Consequently, Monod devoted all of his scientific efforts towards understanding enzymatic adaptation and not inhibition. Now, we ask, why might Lwoff intentionally have altered Monod’s words? We offer three non-exclusive hypotheses. First, Lwoff may have found this scenario from enzymatic adaptation to the operon more straightforward to transmit to scientists and general audiences. This simplified interpretation of Monod’s diauxic studies, however, thus completely changed the meaning: Instead of Monod’s sentence, “Since that day in December 1940, all my scientific activity has been devoted to the study of *this phenomenon*,” Lwoff substituted the term *enzymatic adaptation*. Monod's work would have been more complicated to explain if one had to include his work on enzymatic *inhibition* in addition to that of enzymatic *adaptation*.

The second hypothesis is scientifically based. In his obituary tribute to Monod, Lwoff recognized that for Monod the “diauxic curve showed an inhibition rather than an adaptation” (Lwoff 1977, p. 388). This phrase can mean that Lwoff was fully aware of Monod’s inhibition interpretation. In contrast to Monod, however, Lwoff considered diauxie observations as perfect examples of enzymatic adaptation, as per his statement: “Induced enzyme synthesis was the key to diauxie.” But why was Lwoff so persistent in interpreting diauxie as enzymatic adaptation? We suggest that Lwoff, as Monod’s mentor in 1940, wanted to encourage Monod to continue his studies on enzymatic adaptation rather than enzymatic suppression. Enzymatic adaptation, at this time, was the only convincing model that could be experimentally tested, using the formation of a specific enzyme with the addition of its substrate. This was emphasized by Monod in 1952:

“Enzymatic adaptation” remains thus far the only phenomenon that directly gives rise to experimentation on the ontogeny of enzymes. This is what makes it interesting, and it is from this point of view that we envision it. (Monod and Cohn 1952, p. 67)

The leading group in this field was Marjory Stephenson’s team at Cambridge, in which Yudkin had proposed his model of adaptive enzyme formation he called “mass action theory” (Yudkin 1938). Thus, when Monod presented his results on diauxie to Lwoff, in December 1940 at the Pasteur Institute, Lwoff believed that it was the perfect occasion to use Monod’s results to make major advancements towards understanding enzymatic adaptation research, in competition with Stephenson’s working model. For Lwoff, the specific nature and experimental possibilities offered by the adaptation aspects of diauxie seemed a much more promising research topic to explore, as opposed to the more general, vague, and not well-studied “glucose diauxic inhibition” phenomenon that Monod was beginning to observe, characterize, and wished to further understand.

Our last hypothesis as to why Lwoff changed *phenomenon* to *adaptation* relies on the fact that diauxie was the origin of the Nobel Prize-winning story for Lwoff’s French, Pastorian team. Even though the phenomenon of diauxie is linked to both adaptation and inhibition of enzymes, it was the work on enzymatic adaptation or induction that lead to the 1965 Nobel Prize. Furthermore, Monod’s studies concerning glucose diauxic inhibition were overtaken by his competitor, Magasanik, who interpreted the effect of glucose during diauxie as part of a more general phenomenon, catabolite repression (Magasanik 1961). Thus, Lwoff considered that Monod’s diauxic inhibition work should not be mentioned because it was surpassed by the studies of Magasanik. In addition, assimilating diauxie with enzymatic adaptation also had certain political repercussions concerning Monod’s pedigree. For Lwoff, Monod’s work was part of a French and Pasteurian research tradition on enzymatic adaptation. The famous Pasteurian researchers, Louis Pasteur and Emile Duclaux, who previously worked on enzymatic adaptation, were also, respectively, the first and second directors of the Pasteur Institute. One of Monod's biographers points out the glorious Monod Pasteur pedigree:

According to Monod, the first to have discovered the phenomenon would have been Emile Duclaux. However, before Duclaux, there had been Pasteur himself…. This is exactly what Monod would later rediscover and call "diauxie." (Schwartz 2017, p. 22)

However, if we now consider diauxie with its two aspects, enzymatic inhibition and enzymatic adaptation, no members of this Pastorian lineage before Monod, neither Pasteur, Duclaux, nor Diénert, would have been able to observe the diauxie phenomenon because they did not have access to the proper experimental conditions. In fact, the team that came the closest to using experimental conditions as those of Monod was that of Marjorie Stephenson three years before Monod’s diauxie discovery in 1940 (Stephenson and Gale 1937b). In Lwoff’s account of French Pastorian research that won the Nobel Prize, these national and institutional interests were possibly taken into consideration.

Lwoff's public modification of Monod's Nobel discourse in his obituary tribute to Monod exerted a major influence on subsequent historical accounts. It related Monod’s research on enzyme adaptation with the operon model, but not diauxic inhibition. Even today, the importance of Monod’s research on diauxic inhibition continues not to be fully recognized. Despite Lwoff's insistence that Monod's discovery was a case of enzymatic adaptation, as this article shows, Monod, for his part, never relinquished his passionate interest in diauxic inhibition.
